# Complex Electroresponsive Dynamics in Olivocerebellar Neurons Represented With Extended-Generalized Leaky Integrate and Fire Models

**DOI:** 10.3389/fncom.2019.00035

**Published:** 2019-06-06

**Authors:** Alice Geminiani, Claudia Casellato, Egidio D’Angelo, Alessandra Pedrocchi

**Affiliations:** ^1^NEARLab, Department of Electronics, Information and Bioengineering, Politecnico di Milano, Milan, Italy; ^2^Department of Brain and Behavioral Sciences, University of Pavia, Pavia, Italy; ^3^IRCCS Mondino Foundation, Pavia, Italy

**Keywords:** neuronal modeling, point neuron, neuron model simplification, neuronal electroresponsiveness, olivocerebellar neurons

## Abstract

The neurons of the olivocerebellar circuit exhibit complex electroresponsive dynamics, which are thought to play a fundamental role for network entraining, plasticity induction, signal processing, and noise filtering. In order to reproduce these properties in single-point neuron models, we have optimized the Extended-Generalized Leaky Integrate and Fire (E-GLIF) neuron through a multi-objective gradient-based algorithm targeting the desired input–output relationships. In this way, E-GLIF was tuned toward the unique input–output properties of Golgi cells, granule cells, Purkinje cells, molecular layer interneurons, deep cerebellar nuclei cells, and inferior olivary cells. E-GLIF proved able to simulate the complex cell-specific electroresponsive dynamics of the main olivocerebellar neurons including pacemaking, adaptation, bursting, post-inhibitory rebound excitation, subthreshold oscillations, resonance, and phase reset. The integration of these E-GLIF point-neuron models into olivocerebellar Spiking Neural Networks will allow to evaluate the impact of complex electroresponsive dynamics at the higher scales, up to motor behavior, in closed-loop simulations of sensorimotor tasks.

## Introduction

The variety of neuron types and spiking patterns is thought to play a fundamental role for cerebellar signal processing (Llinás, 1988, 2014) and eventually for motor learning and control. By exploiting pacemaking, bursting, adaptation and more complex properties like oscillation and resonance, cerebellar neurons can precisely encode sensorimotor signals, induce plasticity, filter noise, and efficiently communicate with different cerebellar layers and extra-cerebellar circuits (D’Angelo et al., 2016a).

The electroresponsiveness of cerebellar neurons has been deeply characterized *in vitro* and *in vivo*, allowing to identify, for each neuron type, a set of electrophysiological properties, which can be used as a reference for tuning single neuron models (Table 1). All cerebellar cortical neurons except granule cells show autorhythmic activity that becomes irregular *in vivo* due to synaptic inputs. All cerebellar neurons show an almost linear relationship between input current and firing rate, although with different slopes. In addition, the different cerebellar neurons show specific properties. The Golgi Cells (GoCs) show spike-frequency adaptation (SFA) when depolarized by prolonged currents, post-inhibitory rebound bursts, phase reset, sub-threshold oscillations (STO), and resonance in theta band (Solinas et al., 2007a,b). The granule cells (GRs) exhibit near-threshold oscillations and resonance in theta band (D’Angelo et al., 1998, 2001). The Purkinje Cells (PCs) show a discontinuous *f-I_stim_* curve, hysteresis following current ramp stimulation and bistability emerging with high stimulus currents (intrinsic bursting) (McKay and Turner, 2005; Masoli et al., 2015; Buchin et al., 2016). Intrinsic bursting is characterized by a sequence of bursts (depolarized spiking states) and pauses (hyperpolarized quiescent states), which correlate with burst-pause responses observed *in vivo* during behavior (Loewenstein et al., 2005). PC responses consist of simple and complex spikes: simple spikes are high-frequency regular spikes, generated spontaneously or following Parallel Fiber (PF) activation. Complex spikes consist of a burst of action potentials or spikelets, followed by a pause, resulting from Climbing Fiber (CF) excitation (Miall et al., 1998; Rokni et al., 2009). Molecular Layer Interneurons (MLIs) fire spontaneously with an increased firing irregularity *in vivo* (Lachamp et al., 2009; Jörntell et al., 2010) and have no significant SFA (Galliano et al., 2013). These properties derive from the specific set of ionic channels and from their localization on neuronal dendrites, soma and axons, as well as from the specific nature of synaptic inputs.

**TABLE 1:** Electroresponsive properties of cerebellar neurons.

	**Auto-rhythm (Hz)**	**CV_ISI_ (*in vitro*)**	**f-I_s*tim*_ slope (Hz/pA)**	**SFA**	**Post-inhibitory rebound**	**Phase reset**	**Resonance**	**STO**
**GoC** Forti et al., 2006; Solinas et al., 2007b; D’Angelo et al.,2013	5–15	0.03	∼0.2	✓	✓	✓	✓ (ϑ band)	✓ (ϑ band)
**GR** D’Angelo et al., 2001; Masoli et al.,2017	**–**	**–**	∼4 ÷ 10	–	–	–	✓ (ϑ band)	✓ (ϑ band)
**MLI** Lachamp et al., 2009; Galliano et al.,2013	∼8.5	0.36	∼2.5	–	–	–	–	–
**PC** McKay and Turner, 2005; Molineux et al., 2006; Lennon et al., 2014; Masoli et al.,2015	40–80	0.04	∼0.08	✓	✓	–	–	–
**DCNnL** Llinas, 1988; Aizenman and Linden, 1999; Uusisaari et al., 2007; De Schutter and Steuber,2009	∼30	0.06	∼0.2	✓	✓	–	–	–
**DCNp** Uusisaari et al.,2007	∼10	N.A.	∼0.18	✓	✓	–	–	–
**IO** De Gruijl et al., 2012; Lefler et al.,2013	∼1	–	–	–	✓	✓	✓	✓ (1–7 Hz)

*CV_ISI_, coefficient of variation of inter-spike intervals; SFA, spike-frequency adaptation; STO, sub-threshold oscillations. Reference literature studies are reported in the first column.*

The deep cerebellar nuclei cells (DCNs) express SFA and post-inhibitory rebound bursting, which is fundamental *in vivo* to modulate the motor output (Hoebeek et al., 2010; Uusisaari and Knöpfel, 2011; Ten Brinke et al., 2017). Based on the expression of marker proteins, two major types of DCN neurons have been identified, with different morphologies, electrophysiological properties, and connectivity patterns (Uusisaari et al., 2007). Large non-GABAergic DCNs (DCNnL) mainly project to pre-motor areas, adapting motor commands during learning tasks, while small GABAergic DCNs (DCNp) are connected to the Inferior Olive, providing feedback on the learning process (Uusisaari and Knöpfel, 2011).

The olivocerebellar circuit functioning strongly relies on the complex dynamics of Inferior Olive (IO) neurons. They exhibit a stereotyped response with slow STO undergoing phase-reset after impulse currents (Long et al., 2002; Kazantsev et al., 2004; Choi et al., 2010; Lefler et al., 2013). Following hyperpolarization, IO neurons generate rebound spikes (De Zeeuw et al., 2003), while when a depolarizing input is applied, single somatic action potentials are translated into bursts of axonal spikes at instantaneous frequency that can exceed 400 Hz (Maruta et al., 2007; Mathy et al., 2009). IO bursts elicit PC complex spikes and promote plasticity in the cerebellar cortex.

In this scenario, single neuron properties have been described in detailed models based on multi-compartment neurons for the different cerebellar layers (Solinas et al., 2007b; Steuber et al., 2011; De Gruijl et al., 2012; D’Angelo et al., 2013; Masoli and D’Angelo, 2017). However, representing this rich set of electroresponsive patterns through simplified neuron models is fundamental to develop realistic multiscale Spiking Neural Networks (SNNs). To tackle this issue, we here exploited the Extended-Generalized Leaky Integrate and Fire (E-GLIF) point neuron that allows to model single-point neurons while keeping a realistic picture of multiple essential electrophysiological features such as autorhythm, bursting, adaptation, oscillations, and resonance (Geminiani et al., 2018). The E-GLIF, which was originally used to reproduce the GoC electroresponsiveness (Geminiani et al., 2018), was used here to optimize and test the other cerebellar neurons: GRs, PCs, MLIs, DCNs, and IO. The results shown here are fundamental in view of SNNs simulations where the impact of complex single neuron dynamics will be evaluated at the network and, eventually, at the behavioral level (D’Angelo et al., 2016a).

## Materials and Methods

### Single Neuron Model

To reproduce the firing patterns described in the Section “Introduction,” single neurons were modeled as E-GLIF point neurons. In previous work, E-GLIF proved able to generate the complete set of GoC spiking responses to different inputs, with a minimum number of equations and free parameters. This makes it the best candidate to be used in SNNs to optimize the compromise between biological plausibility and computational load (Geminiani et al., 2018).

Extended-Generalized Leaky Integrate and Fire couples time-dependent with event-driven algorithmic components and includes three linear Ordinary Differential Equations describing the time evolution of membrane potential (*V_m_*) and of two intrinsic currents (*I_adapt_* and *I_dep_*). These three state variables are updated at spike events, which are generated according to a probabilistic threshold crossing.

The model is defined as follows:

$$\{\begin{array}{l} \frac{d\;V_{m}(t)}{\mathrm{dt}}=\frac{1}{C_{m}}\left(\frac{C_{m}}{\tau_{m}}(V_{m}(t)-E_{L})-I_{\mathrm{adap}}(t)+I_{\mathrm{dep}}(t)+I_{e}+I_{\mathrm{stim}}\right)\\ \frac{d\;I_{\mathrm{adap}}(t)}{\mathrm{dt}}=k_{\mathrm{adap}}(V_{m}(t)-E_{L})-k_{2}I_{\mathrm{adap}}(t)\\ \frac{d\;I_{\mathrm{dep}}(t)}{\mathrm{dt}}=-k_{1}I_{\mathrm{dep}}(t) \end{array}$$

Where:

*I_stim_* = external stimulation current;

*C_m_* = membrane capacitance;

τ*_m_* = membrane time constant;

*E_L_* = resting potential;

*I_e_* = endogenous current;

*k_adap_*, *k*_2_ = adaptation constants;

*k*_1_ = *I_dep_* decay rate.

If the neuron is in the refractory period *t_ref_*, spikes cannot be emitted. Otherwise, a spike is generated stochastically at time *t_spk_*, according to an escape rate noise: the nearer *V_m_* is to the threshold potential *V_th_*, the higher the probability to have a spike, depending on an exponential function (Gerstner and Kistler, 2002; Jolivet et al., 2006).

At each spike event, the state variables are updated according to the rules:

$$\{\begin{array}{l} V_{m}\left(t_{\mathrm{spk}}^{+}\right)=V_{r}\\ I_{\mathrm{adap}}\left(t_{\mathrm{spk}}^{+}\right)=I_{\mathrm{adap}}\left(t_{\mathrm{spk}}\right)+A_{2}\\ I_{\mathrm{dep}}\left(t_{\mathrm{spk}}^{+}\right)=A_{1} \end{array}$$

Where:

$t_{\mathrm{spk}}^{+}$ = time instant immediately following the spike time *t_spk_*;

*V*_r_ = reset potential;

*A*_2_, *A*_1_ = update constants of *I_adap_* and *I_dep_*, respectively.

**FIGURE 1:**
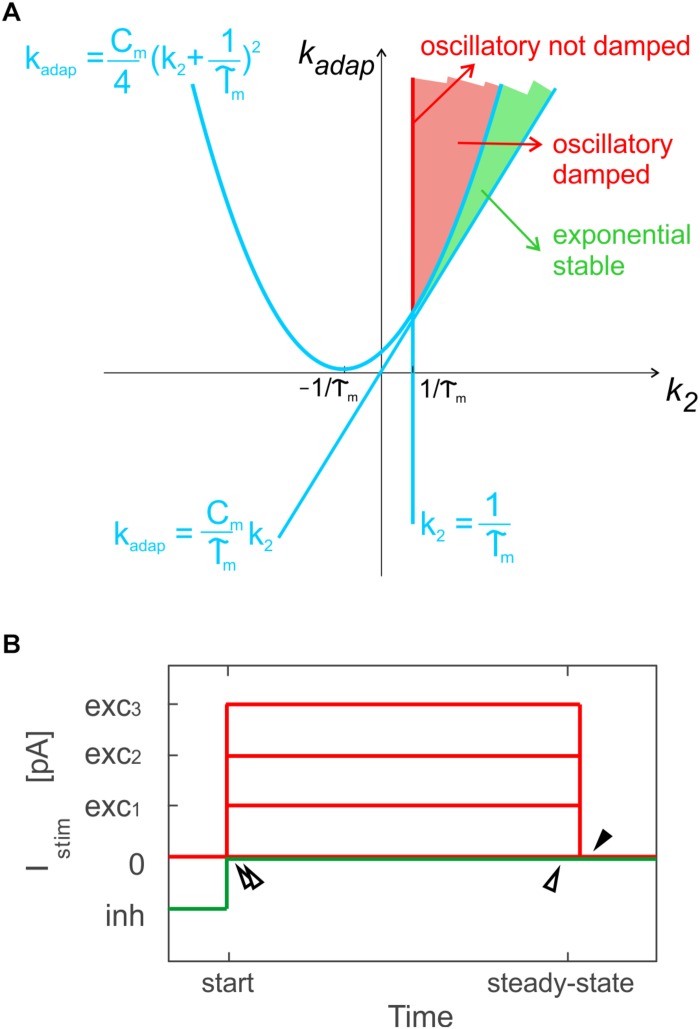
Optimization methods. **(A)** Parameter space for different solution regimes in E-GLIF neuron model, depending on values of parameters *k_adap_* and *k*_2_. The red line corresponds to not-damped oscillatory solutions, the red region to oscillatory damped solutions, while the green area corresponds to exponential stable solutions. Adapted from Geminiani et al. (2018). **(B)** Stimulation protocol for evaluation of model analytical solution used for optimization in specific sub-intervals: time to first spike and between first and second spike, at the beginning of zero-/depolarizing current steps and following a hyperpolarizing step (double white arrows); time between two spikes at steady-state (white arrow); time to first spike (pause) at the end of a strong depolarizing current step, exc_3_, only for PC E-GLIF optimization, to fit the burst-pause response (black arrow).

Based on *k*_2_ and *k_adap_* values, the model exhibits exponential or oscillatory responses (Figure 1A). Elements in the model can be associated to different mechanisms that contribute to the spike patterns. The endogenous current, *I_e_*, accounts for autorhythm and regulation of the intrinsic steady-state membrane potential; the adaptive current, *I_adap_*, coupled with *V_m_* accounts for intrinsic sub-threshold oscillations of the membrane potential and represents the slow hyperpolarizing sub-cellular currents, e.g., the K^+^ channel currents; the spike-triggered current, *I_dep_*, accounts for fast depolarizing mechanisms, e.g., the Na^+^ and low threshold voltage activated Ca^2+^ channel currents. For neuron connections within SNNs, conductance-based synapses are used, with spike-triggered change of synaptic conductance, *g_syn_*, according to an alpha function (Cavallari et al., 2014; Geminiani et al., 2018):

$$g_{\mathrm{syn}}(t)=G_{\mathrm{syn}}\frac{t-t_{\mathrm{spk}}}{\tau_{\mathrm{syn}}}e^{1-\frac{t-t_{\mathrm{spk}}}{\tau_{\mathrm{syn}}}}$$

where *G_syn_* is the maximum conductance change and τ*_syn_* the synaptic time constant.

### Neuron Model Optimization

Analogously to the GoC E-GLIF optimization, for each cerebellar neuron we derived the parameters related to neurophysiological quantities (i.e., *C_m_*, τ*_m_*, *E_L_*, Δ*t_ref_*, *V_th_*, *V_r_*) from literature *in vitro* experiments (Table 2). For the remaining parameters (i.e., *k_adap_*, *k_2_*, *k*_1_, *A*_2_, *A*_1_, *I_e_*), we used the optimization strategy described in Geminiani et al. (2018), developed in MATLAB, where the cost and constraint functions were adapted to consider the electroresponsive properties of each neuron type as in Table 1.

**TABLE 2:** Electrophysiological passive properties chosen from literature for the different cerebellar neurons.

	**C_*m*_ (pF)**	**τ _*m*_ (ms)**	**E_*L*_ (mV)**	**t_*ref*_ (ms)**	**V_*r*_ (mV)**	**V_*th*_ (mV)**
**GoC**	145	44	−62	2	−75	−55
Forti et al., 2006; Solinas et al., 2007a; Tripathy et al., 2014	(145 ± 73)	(44 ± 22)	(−62)	(2 ± 0.4)	(−75)	(−55 ± 1)
**GR**	7	24.15	−62	1.5	−70	−41
D’Angelo et al., 1998, 2001; Tripathy et al., 2014; Houston et al.,2017	(5.5 ± 0.5)	(24.15 ± 2)	(−62 ± 11)	(1.5 ± 0.4)	(−70)	(−41 ± 3)**
**MLI**	14.6	9.125	−68	1.59	−78	−53
Lennon et al., 2014	(14.6)	(9.125)	(−68)	(1.59)	(−78)	(−53)
**PC**	334	47	−59	0.5	−69	−43
Hourez et al., 2011; Hoxha et al.,2012	(334 ± 106)	(47 ± 32)	(−59 ± 6)	(0.5 ± 0.1)	(−69)	(−43 ± 2)
**DCNnL**	142	33	−45	1.5	−55	−36
Uusisaari et al.,2007	(142 ± 31)	(33 ± 18)	(−45 ± 13)	(1.5 ± 0.2)	(−55)	(−36 ± 7)
**DCNp**	56	56	−40	3.02	−55	−39
Uusisaari et al.,2007	(56 ± 26)	(56 ± 30)	(−40 ± 13)	(3.02 ± 0.3)	(−55)	(−39 ± 8)
**IO**	189	11	−45	1	−45	−35
Long et al., 2002; De Zeeuw et al., 2003; Van Der Giessen et al.,2008	(189 ± 12)	(11 ± 4)	(−45)	(1)	(−45)	(−35)

*Experimental reference values are reported in brackets as mean ± SD (Standard Deviation – when available), from literature reference studies reported in the first column.*

#### Optimization Stimulation Protocol

Exploiting the analytical solution of the model, the optimization algorithm aimed at minimizing the error on spike times during three sub-intervals of a current step stimulation period, where the *V_m_* solution could be computed: the time to the first spike, the time between first and second spike and the time between two steady-state spikes (Figure 1B). A multi-step stimulation protocol was considered for optimization, including: a zero-current phase, three phases with increasing depolarizing currents (*exc*_1_ < *exc*_2_ < *exc*_3_), and a zero-current phase following a stimulation interval with a negative current, *inh*.

#### Cost Function

The cost function evaluated the error on the desired spike times (computed from desired output frequency), in order to fit cell-specific quantitative input–output relationships (Supplementary Table S1): (i) autorhythm frequency, when *I_stim_* = 0, (ii) response rates (*freq*_1_ < *freq*_2_ < *freq*_3_), with increasing amplitudes of *I_stim_* (*exc*_1_ < *exc*_2_ < *exc*_3_), and (iii) rebound burst latency and initial frequency, following an inhibitory current step, *inh*. To take into account SFA during depolarizing current steps, the desired steady-state firing rate was obtained from desired frequencies (*freq*_1_ < *freq*_2_ < *freq*_3_) multiplied by an attenuating factor (*factor*_1_, *factor*_2_, *factor*_3_) based on experimental values.

In addition, only PCs exhibit the burst-pause response (Masoli et al., 2015): to account for this specific property, the PC cost function evaluated also the time to the first spike (i.e., the pause), just after the turning off of *I_stim_* = *exc*_3_ (Figure 1B).

#### Optimization Constraints

The cell-specific constraints (Supplementary Table S2) were customized to obtain:

• Neurophysiological ranges of currents in the model;
• Neurophysiological steady-state value of the membrane potential during inhibition (*V_m_inh_*);
• Oscillatory damped or not (red area in Figure 1A) or exponential (green area in Figure 1A) *V_m_* dynamics (Geminiani et al., 2018), based on *k*_2_ and *k_adap_* ranges as in Figure 1A;
• Neurophysiological values of oscillation frequency, in case of oscillatory neurons, i.e., GRs and IOs;
• Sub-threshold value of the steady-state membrane potential (*V_m_ss_tonic_*) and limited amplitude of oscillations (*A_osc_tonic_*) to prevent spontaneous firing in oscillatory neurons without autorhythm – GRs and IOs, in case of zero external input.

The mathematical expression of the cost function, the fitted input-output quantitative patterns and the values of the constraints are reported with proper details in Supplementary Material.

#### Optimization Implementation

For each neuron type, we ran five optimizations with different random initializations of parameters within their ranges, to test the robustness of results with respect to initialization. We chose the optimal parameter set as the median of the final parameters in each optimization run.

### Neuron Model Validation

**FIGURE 2:**
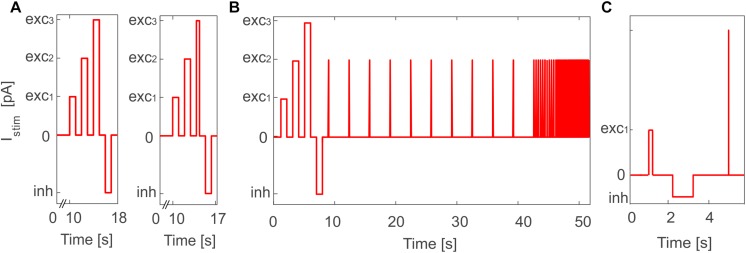
Stimulation protocol for E-GLIF model validation in PyNEST simulations. **(A)** General *in vitro* protocol with the three depolarizing current steps (exc_1,2,3_) and the inhibitory step (inh) used for PyNEST simulations of MLI and DCN E-GLIF (left panel); a shorter exc_3_ current step is used for PCs to test the burst-pause response (right panel). The current amplitude values are the same used in the optimization process, where only sub-intervals of each stimulation phase were considered. **(B)** Customized protocol for GR E-GLIF to test resonance through a stimulation phase with periodic spike trains at increasing frequencies. **(C)** Customized protocol for IO E-GLIF with one shorter depolarizing step and an impulse stimulus to evaluate phase reset of membrane potential oscillations.

To validate the outcome of optimization and test the effective proper functioning of the model based on literature data, we simulated the E-GLIF responses during a continuous stimulation protocol with current steps in PyNEST (Diesmann and Gewaltig, 2002). This validation was fundamental to assess the result of optimization that was based on the evaluation of the neuron response only in sub-sampled intervals of a continuous simulation. In order to evaluate all the electroresponsive properties in Table 1, the stimulation protocol included a first phase with zero external current, where to measure autorhythm and irregular firing, followed by three depolarizing phases lasting 1 s and interleaved with 1-s zero-current intervals, to measure intrinsic excitability and adaptation. Afterward, a 1-s inhibitory current was applied and turned off in the subsequent step, to test rebound bursting (Figure 2A, left panel). The amplitudes of current steps in each phase were the same used during optimization, but the whole continuous response was here assessed, and not just the sub-intervals included in the optimization. The stimulation protocol was then customized with additional or modified phases for neurons with specific electroresponsive patterns:

• For PCs, we reduced the third depolarizing interval from 1 to 0.01 s (Figure 2A, right panel) to test the burst-pause response with high input currents (McKay and Turner, 2005) and evaluate the effect of current pulses (analogous to CF bursts);
• For GRs, we included an additional phase with input current step trains at increasing frequencies (0.3-3-6-9-12-15 Hz), to evaluate resonance (Figure 2B);
• For IOs, we considered only one depolarizing phase lasting 0.05 s, to adapt to literature reference protocols for *in vitro* experiments. Then, we tested the effect of different current amplitudes on burst response properties and we evaluated phase reset of STO, following a current impulse (amplitude = 1 nA, duration = 5 ms), during a zero-current interval lasting 1.5 s (Figure 2C).

We ran 10 simulations for each neuron and computed the mean ± Standard Deviation (SD) of activity parameters (see section “Validation Data Analysis”).

### Validation Data Analysis

Significant parameters were extracted from spiking time instants to evaluate single neuron firing patterns in validation protocols:

• The tonic firing rate, *f_tonic_*, as the inverse of the mean inter-spike interval (ISI), and the coefficient of variation of inter-spike intervals (CV_ISI_) to quantify the irregularity of firing, during the zero-current phase;
• The firing rate, *f*, as the inverse of the mean ISI, during the first three spikes of each depolarizing phase;
• The steady-state firing rate, *f_ss_*, as the inverse of the mean ISI, during the last six spikes of each 1-s depolarizing phase;
• The *f-I_stim_* slope derived from initial responses to the excitatory step currents;
• The SFA gain, computed as the ratio between *f* and *f_ss_*;
• Latency and initial frequency (i.e., inverse of the first burst ISI), measured in the rebound burst after hyperpolarization (*lat_rebound* and *rebound_freq*, respectively). Post-inhibitory activity was considered a rebound burst if *lat_rebound* and *rebound_freq* were lower than the autorhythm ISI and higher than the autorhythm frequency, respectively.

To quantify resonance in GRs, we also computed the response speed as the inverse of the mean spike latency in each resonance step; the values from multiple simulation tests and frequencies were fitted through a smoothing spline in order to obtain the resonance curve (Gandolfi et al., 2013).

## Results

The single-point models of cerebellar neurons were generated using E-GLIF protocol (Geminiani et al., 2018) and were tuned toward their specific neurophysiological response patterns. For GoCs, we used the same optimal parameters reported in Geminiani et al. (2018). For the other neurons, after fixing the passive properties from literature data (Table 2), the optimization algorithm was used to tune the remaining model parameters toward specific electrophysiological features. In most cases, the algorithm converged to the same region of the parameter space over the five optimization runs (Supplementary Figures S1, S2). The resulting parameter sets achieved the optimal compromise between minimum cost function and constraint violation (below 1.0 and 0.1, respectively), best reproducing the electroresponsiveness of each neuron type (Table 3).

**TABLE 3:** Optimized parameter sets of E-GLIF models for each neuron type.

	***k_adap_* (MH^–1^)**	***k*_2_ (ms^–1^)**	**A_2_ (pA)**	**k_1_ (ms^–1^)**	**A_1_ (pA)**	**I_e_ (pA)**
GoC (Geminiani et al., 2018)	0.217	0.023	178.01	0.031	259.988	16.214
GR	0.022	0.041	–0.94	0.311	0.01	–0.888
MLI	2.025	1.096	5.863	1.887	5.953	3.711
PC	1.491	0.041	172.622	0.195	157.622	742.534
DCNnL	0.408	0.047	3.477	0.697	13.857	75.385
DCNp	0.079	0.044	176.358	0.041	176.358	2.384
IO	1.928	0.091	1358.197	0.191	1810.923	–18.101

**FIGURE 3:**
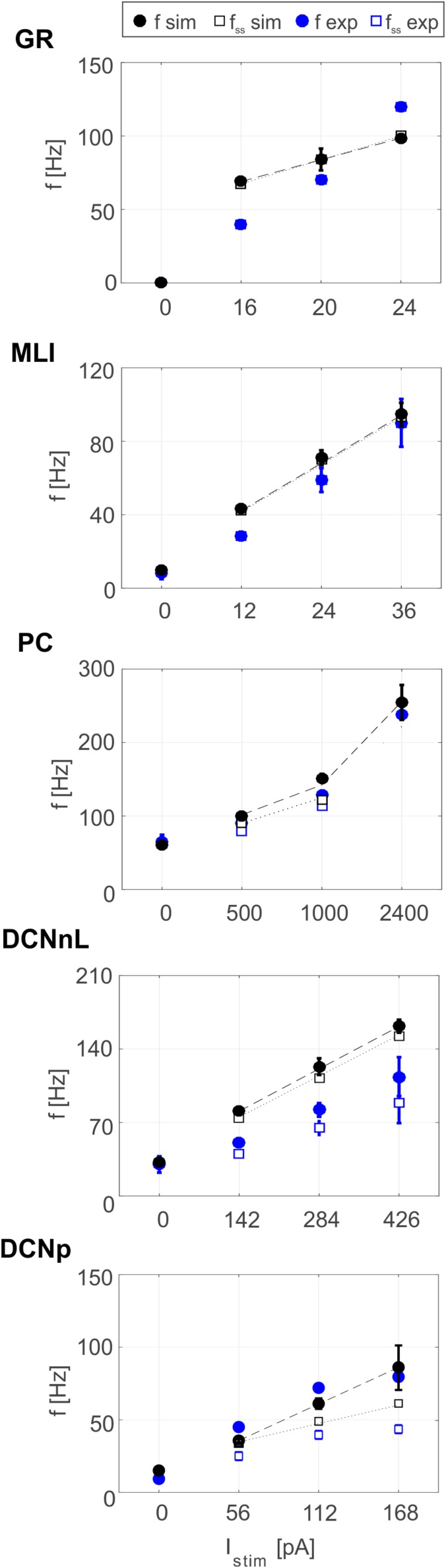
Plots of *f-I_stim_* relationships for GRs, PCs, MLIs, and DCNnL neurons, comparing outcome of PyNEST simulations (black markers) with literature target values used for optimization (blue markers), at the beginning (circles), and after 1-s (squares) current step stimulation. Experimental data taken from D’Angelo et al. (2001), McKay and Turner (2005), Uusisaari et al. (2007), and Galliano et al. (2013).

**FIGURE 4:**
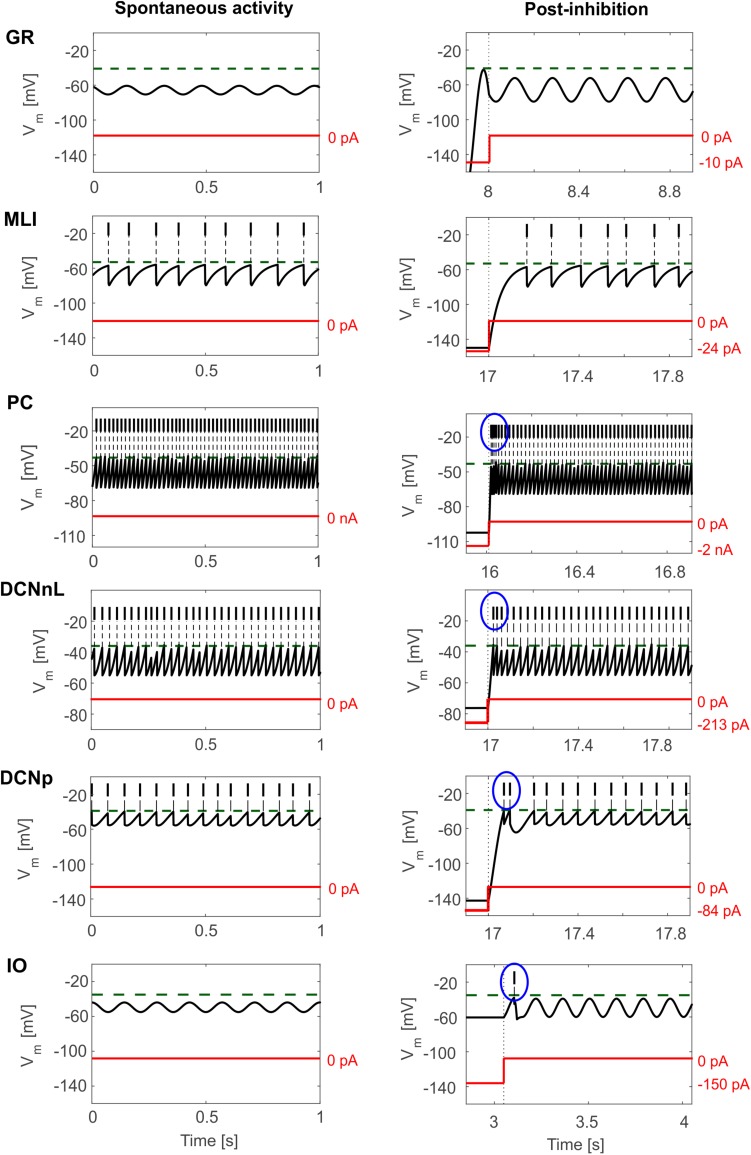
E-GLIF responses to zero-input current (left column) and following a hyperpolarizing current step (right column) for the main olivo-cerebellar neurons. Zero-current inputs cause STO in GR and IO neurons, and autorhythm in the others. Post-inhibitory rebound excitation (burst or spike) is highlighted in the blue circle, where present.

**FIGURE 5:**
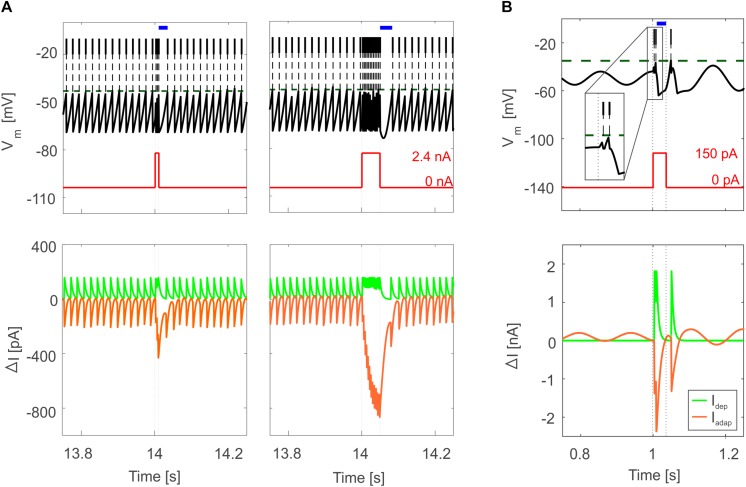
Bursting responses in E-GLIF simulations. **(A)** Burst-pause in PC E-GLIF with a 10-ms input current step (left panel) and 50-ms input current step (right panel). *V_m_* and input current traces are reported in top panels, showing the burst during the stimulation phase and the subsequent pause (blue segment) when the current goes back to 0 nA. Model current traces are reported in bottom panels, with respect to their steady-state value (ΔI). The I_adap_ current is reported in negative values as it has a hyperpolarizing effect in the neuron model. At the end of the stimulation, the accumulated inhibitory effect of I_adap_ causes the pause, until it decays, and the tonic balance of currents is restored. **(B)** Bursting response in IO E-GLIF during a 50-ms current step stimulus, showing a first doublet (zoom in the inset) followed by a pause (blue segment); even in this case, the intrinsic model currents drive the *V_m_* response (bottom panel).

**FIGURE 6:**
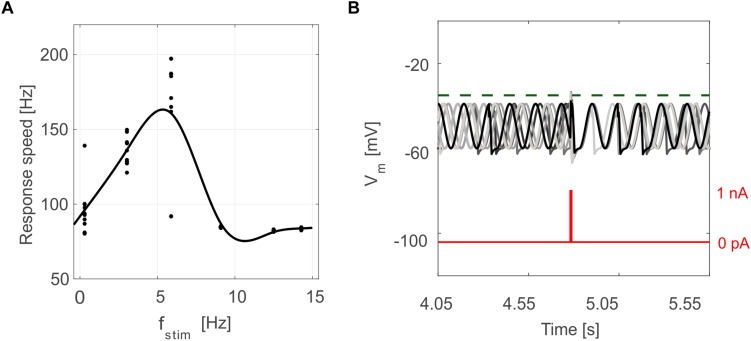
Oscillation-driven properties in E-GLIF simulations. **(A)** Resonance in GR E-GLIF following spike train stimulation at increasing frequencies. The resonance curve was obtained fitting the data points from 10 simulations (black dots) with a smoothing spline. **(B)** Phase-reset of IO E-GLIF *V_m_* during 10 simulations with the same current pulse, causing a spike and a subsequent reset of oscillation phase, independent from the phase before the stimulus.

Tuned E-GLIF neurons were then tested in PyNEST simulations with the stimulation protocol described in the Section “Neuron Model Validation.” The model was able to capture the intrinsic excitability of all neurons, generating linearly increasing firing rates with depolarizing current steps. As shown in Figure 3, frequencies values and *f-I_stim_* slope were close to the target values for all neurons or within acceptable ranges. For GRs the *f-I_stim_* slope was lower than in the reference study (D’Angelo et al., 1998) but still consistent with experimental ranges (Spanne et al., 2014; Masoli et al., 2017). In DCNnL, depolarization frequencies were higher than target values, but linearly increasing with an acceptable *f-I_stim_* slope (Table 4). SFA was present for PCs and DCNnL with average SFA gain of 1.1 at all *I_stim_* values, close to the target values of adaptation gain from electrophysiological recordings (1.1 and 1.2, respectively) (Uusisaari et al., 2007; Kim et al., 2013). In DCNp, SFA was more pronounced, with an average gain of 1.3 for I_stim_ = *exc*_2,3_ (Uusisaari et al., 2007). In absence of external stimuli, PC, MLI and both DCN E-GLIF produced irregular autorhythm at physiological frequencies, while GRs and IOs generated STO at 6 and 7 Hz, respectively (Figure 4). At the end of a hyperpolarizing current step, PCs and DCNs exhibited rebound excitation (doublets/bursts), which is fundamental for efficient signal transmission (Figure 4). In IOs, post-inhibitory rebound spikes were generated with 50% probability, as in experiments (De Zeeuw et al., 2003; Mathy et al., 2009). When stimulating PC with current pulses of 2.4 nA, the typical intrinsic bursting (burst-pause response) was generated. This was achieved thanks to the balance of model currents, *I_dep_* and *I_adap_* that accounted for subcellular mechanisms leading to PC complex spikes (De Zeeuw et al., 2011). A 10-ms pulse caused a burst at 254.58 ± 18.26 Hz followed by a pause of 23.47 ± 2.38 ms, longer than the tonic ISI (Figure 5A); with a 50-ms current step the neuron was silent for 32.46 ± 1.22 ms after a burst at 234.87 ± 2.70 Hz (Figure 5B; Grasselli et al., 2016). This spiking pattern well fits with the PC response to dendritic current injection; however, the typical PC bistable regime caused by a continuous high-amplitude stimulation could not be reproduced in the model without losing other electroresponsive properties (Masoli et al., 2015). Intrinsic STO in GRs lead to resonance at 6 Hz, when stimulating the GR neuron model with periodic spike trains at increasing frequencies (Figure 6A). Finally, the optimized E-GLIF model was able to generate also the typical IO bursting response (193.91 ± 24.58 Hz) in case of current step input, thanks to the rapid effect of *I_dep_* at the beginning of stimulation and the slower accumulation of *I_adap_* that blocked the firing (Figure 5B). Increased amplitudes of the input current caused a non-linear increase of the burst frequency, within physiological ranges; instead, lower currents (i.e., 200 pA) were not sufficient to activate bursts, but they only produced single spikes followed by a pause. Current pulses in the IO E-GLIF induced a spike and a subsequent phase reset of STO, independent from the phase of the stimulus (Figure 6B). Consistently with experimental results, post-impulse STO phase in the model was (0.87 ± 0.02)⋅T for pre-stimulus phases ranging from 0.06⋅T to 0.92⋅T, being T the period of oscillations (Kazantsev et al., 2004; Lefler et al., 2013).

**TABLE 4:** Intrinsic excitability properties of optimized E-GLIF neurons.

	***f_tonic_* (Hz)**	**CV_ISI_ (mean)**	***f-I_stim_* slope**	***lat_rebound* (ms)**	***rebound_freq* (Hz)**
GR	–	–	3.70	–	–
MLI	9.51 ± 0.17	0.13	2.16	(172.96 ± 11.07)	(10.03 ± 1.54)
PC	60.96 ± 0.15	0.04	0.08	10.62 ± 0.15	183.01 ± 6.14
DCNnL	31.48 ± 0.16	0.06	0.28	23.95 ± 0.39	64.81 ± 4.49
DCNp	14.37 ± 0.1	0.09	0.4	69.32 ± 0.94	42.14 ± 3.54
IO	–	–	–	59.22 ± 1.96	–

*Values are reported as mean ± SD over the 10 PyNEST simulations for each neuron type.*

Therefore, the whole set of olivo-cerebellar cells could be modeled with E-GLIF neurons, generating realistic spiking patterns and capturing crucial electroresponsive properties for cerebellar functioning.

## Discussion

In this paper, the E-GLIF model (Geminiani et al., 2018), that was previously developed and validated for Golgi cells, was tuned toward the unique electroresponsive properties of granule cells, Purkinje cells, molecular layer interneurons, deep cerebellar nuclei cells and inferior olivary cells. In these neurons, E-GLIF effectively reproduced pacemaking, adaptation, bursting, post-inhibitory rebound excitation, subthreshold oscillations, resonance, and phase reset. Therefore, for the first time, a whole set of single point neurons is made available to investigate the functional dynamics of the olivocerebellar circuit (Voogd and Glickstein, 1998; Ruigrok, 2011; D’Angelo et al., 2013; Witter et al., 2013; Zhou et al., 2014). These include oscillations and resonance, which are thought to play a critical role for network entraining into large-scale brain oscillations (De Zeeuw et al., 2011; Courtemanche et al., 2013; Llinás, 2014), and long-term synaptic plasticity, which is considered the main mechanism underlying the cerebellar role in motor control and learning (Ito et al., 2014; D’Angelo et al., 2016b).

### Modeled Single Neuron Dynamics

Extended-Generalized Leaky Integrate and Fire (Geminiani et al., 2018) is a simplified point-neuron based on a system of three linear ordinary differential equations and its analytical tractability allows to define different solution regimes and to tune model parameters through a generalizable optimization algorithm. In the current work, E-GLIF was able to simulate complex input-output relationships of cerebellar and IO neurons, generating cell-specific intrinsic excitability and non-linear firing properties that would not be possible using previous GLIF models (Mihalaş and Niebur, 2009).

For neurons with oscillatory *V_m_*, the second order dynamics of the model allowed to simulate intrinsic self-sustained STO. Second order dynamics allowed to reproduce also other non-linear electroresponsive behaviors like resonance in GRs and phase reset of STO in IO neurons. These properties have been measured in single-neuron experiments and are probably amplified at network level (D’Angelo et al., 2001). Specifically, the feedback inhibitory loop from GoCs to GRs is supposed to contribute to resonance and oscillations in the Granular layer network, enhancing theta-band signals coming from extra-cerebellar regions (D’Angelo and Casali, 2013; Gandolfi et al., 2013). Future simulations of the granular layer network with E-GLIF neurons will help to elucidate the different contribution of single cell and circuit properties on network oscillations and resonance. This would extend the results of previous studies where detailed microcircuit models and SNNs with Leaky Integrate-and-Fire units were exploited (D’Angelo et al., 2013; Casali et al., 2019). In the IO circuit, phase reset of STO has been measured in single neurons (Kazantsev et al., 2004), but synchronous stimulation of an olivary area was shown to amplify this response (Lefler et al., 2013). The IO E-GLIF could reproduce the first response during simulation of *in vitro* protocols. In principle, adding gap junctions to the neuron model would account also for the phase-reset amplification at network level, thanks to the intrinsic communication within IO nuclei.

To simulate IO neurons, E-GLIF was optimized taking the axonal bursting regime as the target behavior (Maruta et al., 2007; Mathy et al., 2009). This aspect challenges the traditional view of CFs as a low-frequency all-or-none signaling pathway: indeed, bursting and rebound activity in IO is fundamental for information encoding, as rebound excitation amplifies the feedback from DCNp cells and olivary bursts elicit complex spikes at PC level. PC E-GLIF successfully reproduced regular firing and the burst-pause pattern following dendritic current stimulation *in vitro*, which can be associated to simple and complex spikes *in vivo* (Masoli et al., 2015). However, bistability and spiking patterns with longer bursts and pauses could not be obtained in the E-GLIF model without losing intrinsic excitability properties. For simulations in SNNs, this is a sufficient approximation since it allows to generate the typical PC network spiking patterns, as shown in the Section “Results.” However, for a more detailed representation even of axonal responses, a multi-compartment version of the PC E-GLIF could be implemented, where multiple E-GLIF neurons are optimized to reproduce the electroresponsiveness of the main PC compartments.

In cerebellar nuclei neurons, rebound excitation has been widely proven *in vitro* but long debated *in vivo* (Alviña et al., 2008). However, recent experimental findings demonstrate that rebound bursting correlates with motor responses and is fundamental for integrating synaptic inputs from PCs, MFs, and IO neurons that all converge in the cerebellar nuclei (Hoebeek et al., 2010; Manto and Oulad Ben Taib, 2010; Witter et al., 2013; Sarnaik and Raman, 2018). Rebound excitation also contributes to cerebellum-driven learning, as demonstrated for associative learning (Ten Brinke et al., 2017). Single-neuron rebound properties are thus crucial in SNNs aimed at multiscale simulations of sensorimotor tasks.

This scenario shows the capability of the E-GLIF point neuron to reproduce the variety of olivo-cerebellar spiking responses following different input stimuli, through a single optimal set of model parameters. Conversely, the traditional approach for single neuron modeling aims at identifying different regions of the parameter space corresponding to different spiking behaviors (Izhikevich, 2003). This makes E-GLIF a best candidate for simulations of SNNs, where neuron response needs to depend on the received input, rather than on the parameter values, achieving higher neurophysiological realism without increasing computational load.

## Conclusion

The E-GLIF single-point neuron models were able to capture the complex non-linear dynamics of olivocerebellar neurons including spontaneous firing, subthreshold oscillations, bursting, phase-reset, and resonance. These ingredients, coupled to algorithms accounting for synaptic integration over dendrites (e.g., Marasco et al., 2012; Rössert et al., 2016), will provide the fundamental ingredients to reconstruct non-linear dynamics in extended spiking cerebellar networks. Future work will include embedding these neuron models into cerebellar SNNs to simulate cerebellum-driven motor paradigms and evaluate the impact of single neuron electroresponsiveness on network dynamics, plasticity and, eventually, motor behavior.

## Data Availability

All datasets generated for this study are included in the manuscript and/or the Supplementary Files.

## Author Contributions

AG and CC elaborated the mathematical model and optimization, designed and carried out the simulations for each neuron, performed the data analysis, and wrote the manuscript. ED and AP coordinated the whole work and substantially contributed to the writing of the final manuscript.

## Conflict of Interest Statement

The authors declare that the research was conducted in the absence of any commercial or financial relationships that could be construed as a potential conflict of interest.
