# Corrigendum: Complex Electroresponsive Dynamics in Olivocerebellar Neurons Represented With Extended-Generalized Leaky Integrate and Fire Models

**DOI:** 10.3389/fncom.2019.00048

**Published:** 2019-07-19

**Authors:** Alice Geminiani, Claudia Casellato, Egidio D'Angelo, Alessandra Pedrocchi

**Affiliations:** ^1^NEARLab, Department of Electronics, Information and Bioengineering, Politecnico di Milano, Milan, Italy; ^2^Department of Brain and Behavioral Sciences, University of Pavia, Pavia, Italy; ^3^IRCCS Mondino Foundation, Pavia, Italy

**Keywords:** neuronal modeling, point neuron, neuron model simplification, neuronal electroresponsiveness, olivocerebellar neurons

In the published article, there was an error regarding the affiliations for “Egidio D'Angelo.” As well as having affiliation 2, he should also have IRCCS Mondino Foundation, Pavia Italy. The authors apologize for this error and state that this does not change the scientific conclusions of the article in any way. The original article has been updated.

