# Supplementary material for: Complex Electroresponsive Dynamics in Olivocerebellar Neurons Represented With Extended-Generalized Leaky Integrate and Fire Models
Source: Front Comput Neurosci. 2019 Jun 6;13:35. doi: 10.3389/fncom.2019.00035 (PMC6563830; doi:10.3389/fncom.2019.00035)
Supplement: Supplementary file 1 [file Data_Sheet_1.docx]

Complex electroresponsive dynamics in olivocerebellar neurons represented with Extended-Generalized Leaky Integrate and Fire models

The neuron model code and the simulation data can be found in the Brain Simulation Platform at https://www.humanbrainproject.eu/en/brain-simulation/brain-simulation-platform/.

Supplementary material: single-neuron optimization

**Cost function**

E-GLIF optimization was based on a gradient-descend algorithm, which optimizes the spike times in sub-intervals of a current-step stimulation protocol to reproduce electrophysiological input-output patterns (Geminiani et al., 2018). To this aim, cell-specific values of input currents (I_stim_), and corresponding desired output frequencies were considered, based on literature data (Table S1): the autorhythm frequency, tonic_freq, with I_stim_ = 0 pA; the depolarization frequencies (f = freq_1_ < freq_2_ < freq_3_) and their steady-state value (according to an attenuation factor, factor_1_,  factor_2_,  factor_3_), with I_stim_ = exc_1_ < exc_2_ < exc_3_; the rebound properties, i.e. time of the first spike – lat_rebound, and initial frequency – rebound_freq, with I_stim_ = 0 pA after a hyperpolarizing phase with I_stim_ = inh. For each current step, the model solution was computed during specific sub-intervals: the time to the first spike – Δt_1_, the time between first and second spike – Δt_2_, and the time between two steady-state spikes – Δt_SS_.

$$cost\_function=\frac{\sqrt{\sum{error}_{\left( i \right)}+{error}_{\left( inh \right)}}}{6} \left( i \right)=0 pA, exc1, exc2, exc3$$

Where:

$${error}_{(i)}=\frac{1}{3}\cdot\left[ \left( {error\_\Delta t}_{1}^{\left( i \right)} \right)^{2}+ \left( {error\_\Delta t}_{2}^{\left( i \right)} \right)^{2}+ \left( {error\_\Delta t}_{SS}^{\left( i \right)} \right)^{2} \right]$$

is the error in each one of the three sub-intervals *Δt_1_*, *Δt_2_*, *Δt_SS_*, with each current step value *I_stim_* = *(i)* = *0 pA*, *exc_1_*, *exc_2_*, *exc_3_*.

$${error}_{(inh)}= \left( {error\_burst\_\Delta t}_{1}^{\left( i \right)} \right)^{2}+ \left( {error\_burst\_\Delta t}_{2}^{\left( i \right)} \right)^{2}$$

is the error in the first two sub-intervals (*Δt_1_*, *Δt_2_*) of a zero-current step, following a hyperpolarizing step with *I_stim_* = *inh*. This term allows to evaluate the properties of rebound bursting, i.e. latency and initial frequency.

Only for PCs, the term ${error\_\Delta t}_{SS}^{\left( i \right)}$, for *(i)* = *exc_3_*, was substituted with the error on the time to the first spike after the end of the depolarizing step, *error_pause^exc3^,* in order to fit the PC burst-pause response with strong excitatory inputs (Masoli et al., 2015):

$${error}_{exc3}=\frac{1}{3}\cdot\left[ \left( {error\_\Delta t}_{1}^{exc3} \right)^{2}+ \left( {error\_\Delta t}_{2}^{exc3} \right)^{2}+ \left( {error\_pause}^{exc3} \right)^{2} \right]$$

***Optimization constraints***

At the same time, the optimization algorithm took into account mathematical and neurophysiological constraints on tuned parameters, as reported in Table S2 for each olive-cerebellar neuron.

Specifically, model current parameters, i.e. *I_e_*, *A_1_*, *A_2_*, were constrained within neurophysiological values, while *I_e_*, *k_adap_* and *k_2_* were limited to obtain a neurophysiological steady-state value of the membrane potential during inhibition (*V_m_inh_*), based on the model solution as reported in (Geminiani et al., 2018), Equ. A8-A9. In addition, to account for different *V_m_* dynamics (i.e. exponential, oscillatory damped, oscillatory not damped), *k_2_* and *k_adap_* were constrained based on the considerations in Figure 1A. Therefore, to obtain not damped oscillatory *V_m_*, *k_2_* was set to $\frac{1}{\tau_{m}}$ . For neurons with self-sustained *V_m_* oscillations and no autorhythm, i.e. GRs and IOs, further constraints were included, in order to obtain: (i) neurophysiological values of oscillation frequency; (ii) sub-threshold value of the steady-state membrane potential (*V_m_ss_tonic_*) and limited amplitude of oscillations (*A_osc_tonic_*), thus preventing spontaneous firing in case of zero external input. It should be noted that in this case, negative values of the endogenous current *I_e_* were allowed to decrease *V_m_ss_tonic_* ((Geminiani et al., 2018) - Equ. A9) and maintain oscillations below the firing threshold.

***Optimization results***

Optimization aimed at minimizing the cost function, while fulfilling the constraints. Following the rules described in previous paragraphs, parameters converged to the same region of the parameter space for most of E-GLIF neurons, during the 5 optimization runs (Figure S1, S2). This demonstrated the robustness of the optimization algorithm, with respect to different values of starting parameters. For GR and IO E-GLIF that have oscillatory V_m_, only 5 parameters were tuned, as k_2_ was fixed to $\frac{1}{\tau_{m}}$ (cf. previous paragraph).

**Table S1** Electroresponsive properties and corresponding input-output quantitative patterns used for cerebellar neuron optimization. Reference literature studies are reported in the first column.

***Supplementary tables and figures***

|  | **Autorhythm** | **f-I_stim_ relationship** | | **Rebound excitation** | |
| --- | --- | --- | --- | --- | --- |
|  | ***tonic_freq*** | ***I_stim_ = [exc_1,_ exc_2,_ exc_3_]*** | ***f = [freq_1,_ freq_2,_ freq_3_]***  ***(factor_1_, factor_2_, factor_3_)*** | ***inh*** | ***lat_rebound;***  ***rebound_freq*** |
| ***GR***  *(D’Angelo et al., 1998)* | - | [16, 20, 24] pA | [40±1, 70±1, 120±1] Hz  (1, 1, 1) | -10 pA | - |
| ***MLI***  *(Galliano et al., 2013)* | 8.5±2.7 Hz | [12, 24, 36] pA | [30±1, 60±5, 90±10] Hz  (1, 1, 1) | -24 pA | - |
| ***PC***  *(McKay and Turner, 2005)* | 65±7 Hz | [500, 1000, 2400] pA | [90±1, 130±1, 242±1] Hz  (1.1, 1.1, -) | -2000 pA | ≤ 31 ms  ≥ 130 Hz |
| ***DCNnL***  *(Uusisaari et al., 2007)* | 30±6 Hz | [142, 248, 426] pA | [50±2, 80±5, 110±15] Hz  (1.2, 1.2, 1.2) | -213 pA | ≤ 66 ms  ≥ 60 Hz |
| ***DCNp***  *(Uusisaari et al., 2007)* | 10±1 Hz | [56, 112, 168] pA | [25±2, 40±2, 45±2] | -84 pA | ≤ 200 ms  ≥ 20 Hz |
| ***IO***  *(De Zeeuw et al., 2003; Mathy et al., 2009)* | - | [300, -, -] pA | [273±43, -, -] Hz  (5, -, -) | -150 pA | 20±2 ms  100±10 Hz |

**Table S2** Customized constraints for the optimization of each cell-specific E-GLIF.

|  | **Model currents constraints** | **V_m_inh_ range** | **Solution type** | **Oscillation limits** |
| --- | --- | --- | --- | --- |
| ***GR*** | -5 < I_e_ < 5 pA  -10 < A_2_ < 30 pA  0.01 < A_1_ < 30 pA | - | oscillatory | 3 < *f_osc_* < 8 Hz  *V_m_ss_tonic_* < 0.9·*E_L_*  *A_osc_tonic_* < 10 mV |
| ***MLI*** | 0.01 < I_e_ , A_2_, A_1_ < 10 pA  A_2_ < A_1_ | -150 < *V_m_inh_* < -80 mV | exponential | - |
| ***PC*** | 0.01 < I_e_ , A_2_, A_1_ < 1500 pA  A_2_ < A_1_ | -175 < *V_m_inh_* < -45 mV | oscillatory damped/exponential | - |
| ***DCNnL*** | 0.01 < I_e_ < 100 pA  0.01 < A_2_, A_1_ < 500 pA  A_2_ < A_1_ | -150 < *V_m_inh_* < -40 mV | oscillatory damped/exponential | - |
| ***DCNp*** | 0.01 < I_e_ < 100 pA  0.01 < A_2_ < 200 pA  0.01 < A_1_ < 200 pA | -155 < *V_m_inh_* < -60 mV | oscillatory damped/exponential | - |
| ***IO*** | -30 < I_e_ < -5 pA  0.01 < A_2_ < 1500 pA  0.01 < A_1_ < 2000 pA | - | oscillatory | 3 < *f_osc_* < 7 Hz  1.5·*E_L_* < *V_m_ss_tonic_* < *E_L_*  *A_osc_tonic_* < 10 mV |


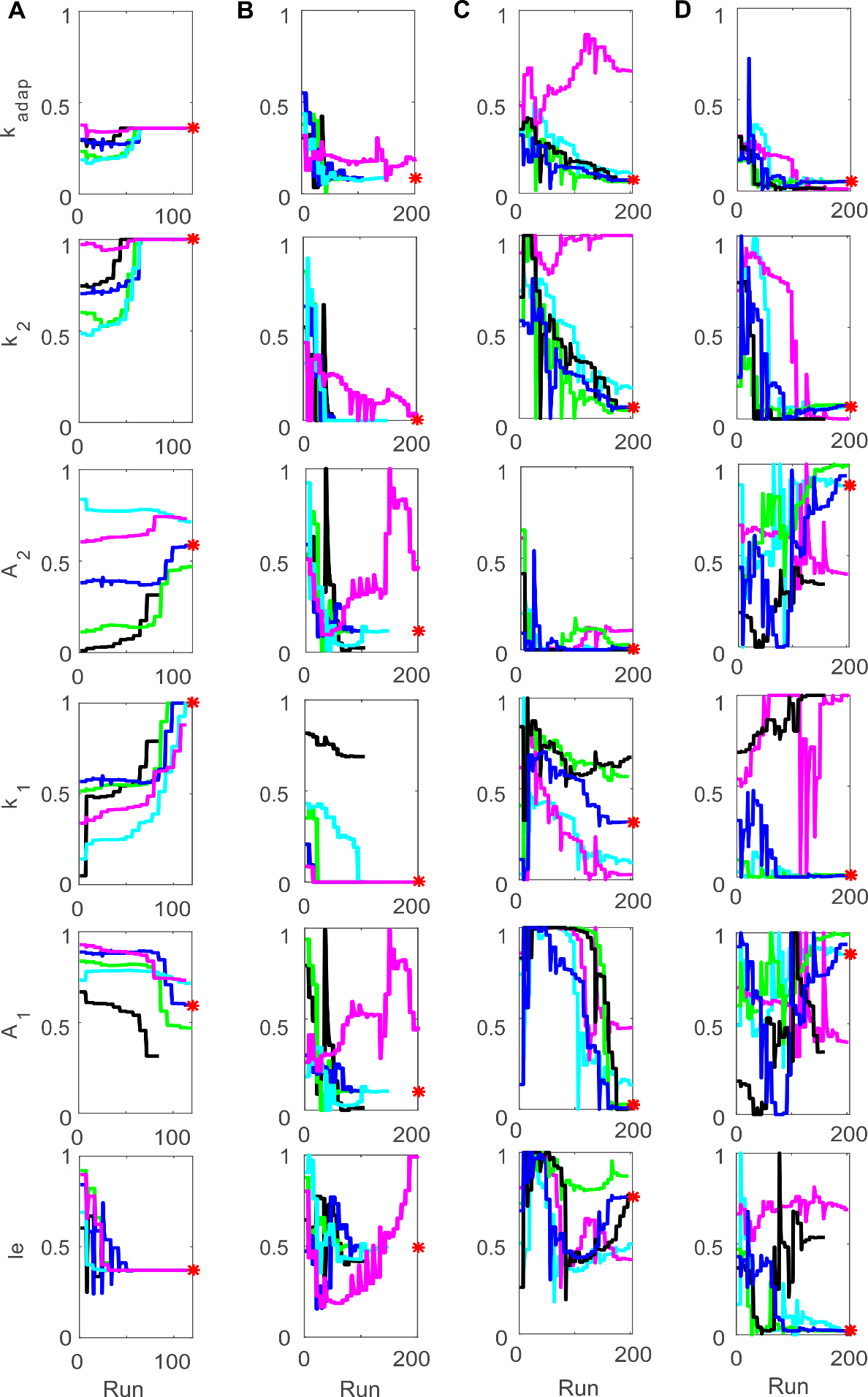


**Figure S1** Normalized parameter values through 5 optimization runs (different colors), for neurons with exponential/oscillatory damped solution: MLI (A), PC (B), DCNnL (C), DCNp (D). The red star represents the final chosen value for each parameter.


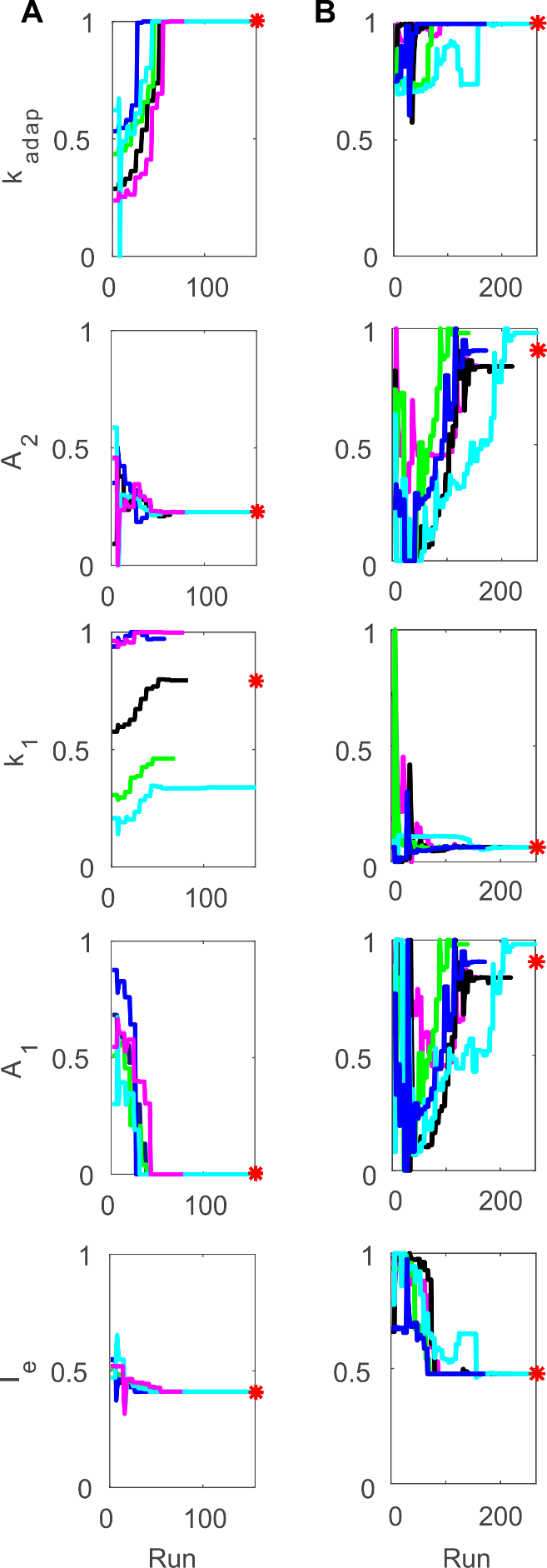


**Figure S2** Normalized parameter values through 5 optimization runs (different colors), for neurons with oscillatory solution: GR (A), IO (B). The red star represents the final chosen value for each parameter. Differently from Figure S1, here k_2_ is not reported, being set to a constant value for obtaining an oscillatory solution.
